# Declines in occurrence of plants characteristic for a nutrient‐poor meadow habitat are partly explained by their responses to nutrient addition and competition

**DOI:** 10.1002/ece3.7306

**Published:** 2021-03-07

**Authors:** Stefanie Höckendorff, Markus Peintinger, Felicitas Fiedler, Marc Stift, Mark van Kleunen

**Affiliations:** ^1^ Ecology, Department of Biology University of Konstanz Konstanz Germany; ^2^ Arbeitsgruppe Bodenseeufer (AGBU) Radolfzell Germany; ^3^ WSL Swiss Federal Research Institute Birmensdorf Switzerland; ^4^ Zhejiang Provincial Key Laboratory of Plant Evolutionary Ecology and Conservation Taizhou University Taizhou China

**Keywords:** eutrophication, grassland, habitat loss, local extinction, *Molinia* meadow, threatened species

## Abstract

Species losses and local extinctions are alarmingly common, frequently as a consequence of habitat destruction. Nevertheless, many intact habitats also face species losses, most likely due to environmental changes. However, the exact drivers, and why they affect some species more than others in apparently intact habitats, are still poorly understood. Addressing these questions requires data on changes in occurrence frequency of many species, and comparisons of the responses of those species to experimental manipulations of the environment. Here, we use historic (1911) and contemporary (2017) data on the presence–absence of 42 plant species in 14 seemingly intact *Molinia* meadows around Lower Lake Constance to quantify changes in occurrence frequency. Then, we performed a common‐garden experiment to test whether occurrence frequencies in 1911 and changes therein by 2017 could be explained by responses of the 42 species to nutrient addition and competition with the acquisitive generalist grass *Poa pratensis*. Within the 14 still intact *Molinia* meadows, 36 of the 42 species had declined since 1911. As expected, nutrient addition generally led to increased biomass production of the 42 target species, and competition with *P. pratensis* had a negative effect. The latter was stronger at high nutrient availability. The more frequent species were in 1911 and the more they declined in frequency between 1911 and 2017, the less above‐ground biomass they produced in our experiment. Competition with *P. pratensis* magnified this effect. Our work highlights that environmental change can contribute to local extinction of species in otherwise intact habitat remnants. Specifically, we showed that increased nutrient availability negatively affected formerly widespread *Molinia*‐meadow species in competition with *P. pratensis*. Our study thus identified a likely mechanism for the decline in occurrence frequency of species in the remaining *Molinia* meadows.

## Graphical abstract

Historic and contemporary presence–absence data was used to quantify changes in occurrence frequency of 42 plant species. To identify whether nutrient addition and competition with an aquisitive grass species drove plant species declines in intact sites of a nutrient‐poor meadow habitat type, a common‐garden experiment with the same 42 plant species was carried out. In competition, increased nutrient availability negatively affected formerly widespread plant species in the experiment.

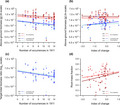

## 1 INTRODUCTION

Biodiversity loss, due to global and local species extinctions, is a worldwide problem for different ecosystems and for different groups of organisms (Ceballos et al., 2015; IPBES, 2019; Régnier et al., 2015). Although many factors contribute to extinctions simultaneously, there is general consensus that the recent rates of global and local species losses are primarily caused by human activities (Bauer et al., 2019; Bowler et al., 2019; Vitousek et al., 1997). Although it is often difficult to attribute species declines to single causes, natural habitat alteration and loss is among the key factors driving local extinction of species (Maxwell et al., 2016; Tilman et al., 1994). Preventing habitat loss is therefore a key priority in conservation, but even protection and management of habitat remnants cannot fully prevent further species losses. Reasons for this could be that the populations in these remnants have become small and genetically isolated making them vulnerable to environmental stochasticity (Aguilar et al., 2008; Honnay & Jacquemyn, 2007; Menges, 1992). In addition, seemingly intact habitat remnants could be compromised by globally acting environmental changes such as increased nutrient loads (e.g., atmospheric nitrogen deposition; Midolo et al., 2019) and climate warming (Steinbauer et al., 2018; Vitousek, 1994).

Plant species that are rare and endangered are often the focus of studies trying to determine the causes of population declines (e.g., Fischer & Matthies, 1997; Noël et al., 2011; Peintinger, 2011, 2012; Prati et al., 2016). However, to better understand why some plant species have declined and others have not, we need comparative studies on multiple species that vary in their extent of decline or that have even increased (e.g., Fischer et al., 2010). Moreover, it is important to distinguish between rare species that used to be more common (i.e., declined recently) and those that have been rare for a long time (Huenneke, 1991). While some studies have compared rare with common species in terms of biomass production (Dawson et al., 2012; Kempel et al., 2020; Zhang & van Kleunen, 2019) and reproductive attributes (Bevill & Louda, 1999; Lavergne et al., 2004; Murray et al., 2002; Zhang & van Kleunen, 2019), few have made similar comparisons between species that differ in how their occurrence frequency changed over time (Fischer & Stöcklin, 1997; Laanisto et al., 2015). Moreover, many studies on rarity compared species irrespective of their habitat affiliations (but see Fischer & Stöcklin, 1997; Stöcklin & Fischer, 1999) and therefore cannot distinguish between species that are rare or declining because their habitats are rare or declining, and species that are declining within their habitats. The latter requires habitat‐specific studies.

Here, we used the *Molinia* meadows along the c. 87 km long shoreline of Lower Lake Constance (Germany and Switzerland) as a model to understand potential drivers of change in occurrence of plant species within a single habitat type. These *Molinia* meadows developed on nutrient‐poor substrates in the 19th century as a result of annual mowing to collect hay for stables (Peintinger, 2012). With the cessation of the mowing practice and due to land‐use change in the last century, many *Molinia* meadows have disappeared. The meadows that remain today are protected and constitute one of the most plant species‐rich habitats in the Lower Lake Constance region (Lang, 1973; Peintinger, 2012). Despite their protected status and the restored annual mowing, the *Molinia* meadows are, like many other habitats around the world, still subject to global environmental changes. For example, the overall deposition rate of atmospheric nitrogen has sharply increased globally in recent decades (Galloway et al., 2008). Furthermore, at the regional scale, flooding events during the eutrophic phase of Lake Constance between the early 1970s and the early 1990s (Jochimsen et al., 2013) have likely led to an additional increase of nutrients such as nitrogen and phosphorous. Although there are no data on the degree of nutrient enrichment of the *Molinia* meadows around Lower Lake Constance, increasing dominance of species typical for nutrient‐rich habitats was documented in similar plant communities in calcareous fens (Caricion davallianae) in Switzerland in recent decades (Bergamini et al., 2009; Moradi et al., 2012; Rion et al., 2018). Indeed, many species have declined in the *Molinia* meadows around Lower Lake Constance (Peintinger, 2012). Like in many other places, this might be due to suppression of those species by dominant grasses that take more advantage of increased nutrient inputs (Aerts & Berendse, 1988; Aerts et al., 1990; Aerts & Bobbink, 1999; You et al., 2017).

The *Molinia* meadows of Lower Lake Constance offer the unique opportunity to study plant species that vary in their historic and current occurrence frequencies, as Baumann (1911) surveyed the species typical for those meadows at the beginning of the 20th century. We had repeated this survey, and thus have unique data on which plant species had low or high occurrence frequencies one century ago, and how this has changed since then (also see Peintinger, 2012). To test how occurrence frequency in 1911 and changes therein relate to the species' responses to increased nutrient availability and to competition, we set up a large outdoor pot experiment. We grew 42 species characteristic of *Molinia* meadows (i.e., habitat specialists) with or without competition with the generalist grass *Poa pratensis* at two levels of nutrient addition. We addressed the following specific main questions: (a) Are the species that benefit least from nutrient enrichment the ones with relatively high occurrence frequencies in 1911 and/or that showed the strongest declines in occurrence frequency by 2017? (b) Are the species that suffer the most from competition the ones with relatively high occurrence frequencies in 1911 and/or that showed the strongest declines in occurrence frequency by 2017? (c) Does nutrient enrichment aggravate the negative impact of competition?

## 2 MATERIALS AND METHODS

### 2.1 Data on changes in species occurrence frequency

**FIGURE 1:**
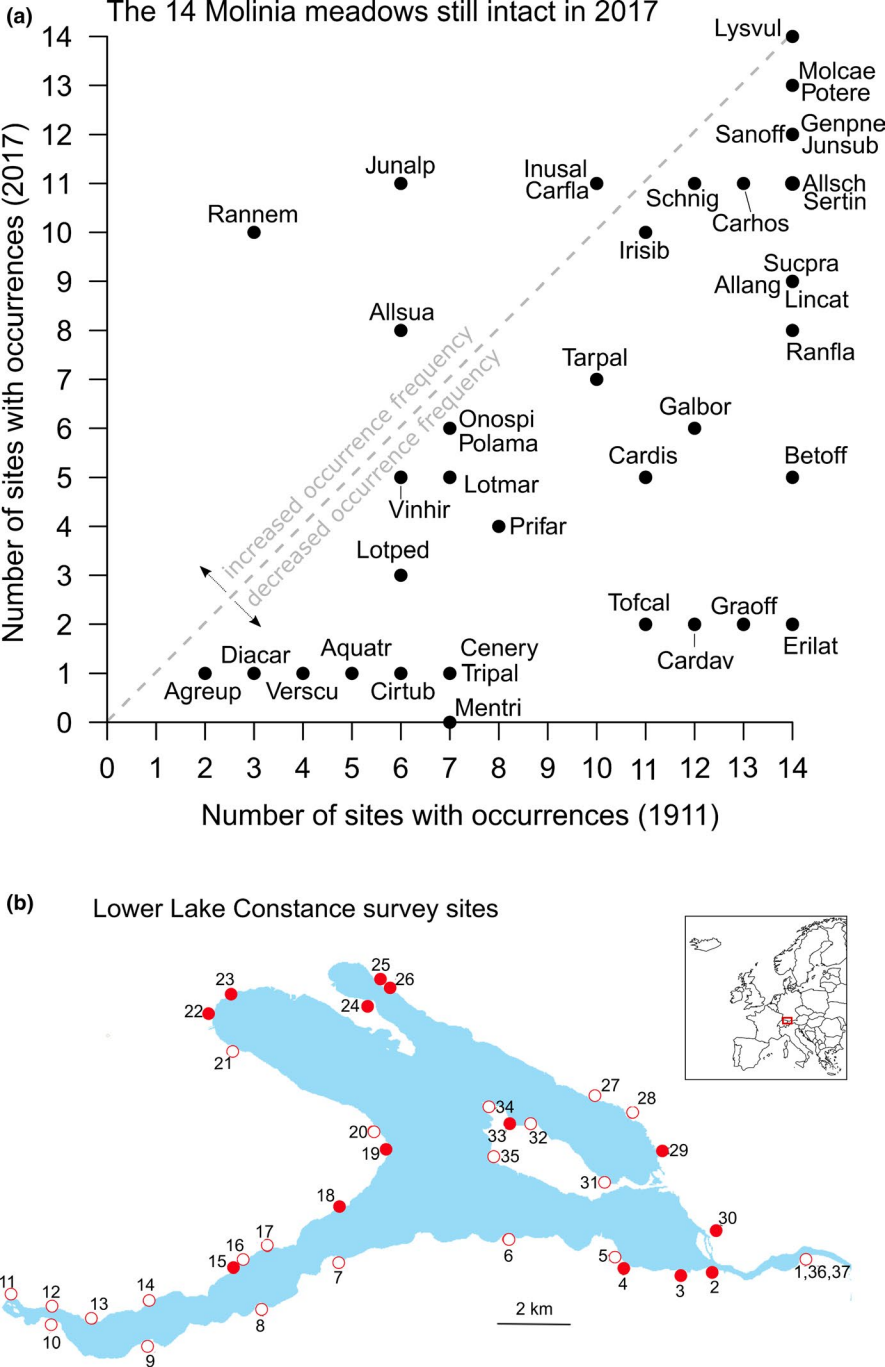
(a) Changes in occurrence frequency of the 42 *Molinia*‐meadow species used in our experiment between 1911 and 2017 in the 14 sites with still intact *Molinia* meadows. (b) Map of Lower Lake Constance and the locations of 37 sites with *Molinia* meadows in 1911, and the 14 still intact ones (filled circles). The inlay shows its location in Europe. More site‐specific information is given in Table S1. Source of lake outline: LUBW (https://udo.lubw.baden‐wuerttemberg.de/public/q/kEeb2; accessed on 1 June 2020)

Based on over 200 excursions between 1905 and 1910, Eugen Baumann published plant species lists for sites around Lower Lake Constance (Baumann, 1911). He aimed for a comprehensive description of the regional flora but did not score the abundances or population sizes of each species. Among others, the presence lists include 91 species that are considered habitat specialists for *Molinia* meadows (i.e., the plant community alliances Molinion caeruleae and Caricion davallianae, according to the Braun‐Blanquet system; Oberdorfer et al., 2001). Peintinger (2012) adapted Baumann's species lists to a presence–absence matrix for the 91 *Molinia*‐meadow species and 37 sites with *Molinia* meadows in the early 1900s (Figure 1b; Table S1). Based on biotope classifications of the State Baden‐Württemberg (LUBW, 2020) and vegetation‐relevé data (Lang, 1973), we a priori classified the current state of the *Molinia* meadows in these 37 sites as being still intact for 14 sites (11 in Germany and three in Switzerland; Table S1). In this study, we focus on species occurrences and changes therein for those 14 sites.

In May and June 2017, we revisited the sites to assess the current occurrence frequencies of the 91 *Molinia*‐meadow species. At each site, we systematically surveyed the whole area for the presence of the 91 species. Like Baumann in the early 20th century, we did this by walking transects through the vegetation. We repeated the surveys in July and August 2017 to verify that we had not missed any species in the spring surveys. Depending on species richness and surface area, a survey generally took up to 3 hrs per site. A total of 25 field days were needed to investigate the 14 intact sites. To rule out that we overlooked some species and because some species might have been absent temporarily (e.g., due to a local flooding in the preceding year), we cross‐checked our own species lists against species lists that were available for the 11 intact sites in Germany through the rare‐plant‐monitoring scheme of the German nature conservation society Naturschutzbund Deutschland (NABU). Only in one of the 11 German sites, the NABU monitoring data listed three species that we had not recorded (we added these), but otherwise our species lists were complete for the 42 species used in our experiment (see below).

### 2.2 Seed collection and precultivation of target species

For 54 of the 91 *Molinia*‐meadow species (representing 28 families; Table S2), we managed to collect seeds in the field or acquire them from other sources for use in our common‐garden experiment. For 40 of those species, the seeds were collected from the 14 intact Lower Lake Constance *Molinia* meadows, and some from the nonintact sites. For seven species, for which seed material from our study sites was unavailable or limited, we additionally collected seeds from other sites in the same region, and in eight cases we obtained seeds from botanical garden collections (Table S2).

Prior to sowing in spring 2018, to break potential seed dormancy, we kept all seeds in a −14°C freezer for 4 weeks, and we soaked seeds of *Cladium mariscus*, *Lotus pedunculatus*, *Lysimachia vulgaris* and *Polygala amarella* in water for 3–4 days. To maximize overlap among species in the date of germination, we sowed species that were anticipated to need more time for germination on 30 April 2018, and the other species 1 week later (Table S2). For each of the 54 species, we sowed seeds from 3–20 seed families, depending on availability (Table S2), on moistened potting substrate (Patzer Einheitserde^®^ CL P, Gebrüder Patzer GmbH & Co. KG) in 7 × 7 × 6.5 cm^3^ pots (TEKU VQB^®^, Pöppelmann). Pots were randomly allocated to positions in a phytochamber of the Botanical Garden of the University of Konstanz with day/night temperatures of 21/17°C, an air humidity of 90% and a 9‐hr day length. The positions of the pots were re‐randomized every 2 weeks.

As 12 of the 54 sown species did not germinate, or took too long to germinate, our experiment included 42 target species from 22 families (Tables S2 and S3). Of these, 36 species had declined (to various degrees) in their occurrence frequency in the 14 intact *Molinia* meadows, while one species (*L. vulgaris*) had remained stable (Figure 1a). The five species that had increased were *Ranunculus nemorosus* (+7 sites), *Juncus alpinus* (+5 sites), *Allium suaveolens* (+2 sites), *Carex flava* (+1), and *Inula salicina* (+1 site). So, our 42 target species covered a wide range of changes in occurrence frequency.

### 2.3 The competitor species

As we hypothesized that many of the characteristic *Molinia*‐meadow species have declined due to increased competition with grasses that have become more dominant, we chose one of those grasses as competitor species. Initially, we intended to use *Molinia caerulea* as competitor, but its seeds failed to germinate in sufficient numbers. Therefore, we instead used *P. pratensis*, another typical acquisitive perennial grass which profits from nutrient enrichment in calcareous, nutrient‐poor grasslands (Fischer & Stöcklin, 1997). The species is native to the Northern Hemisphere and has become naturalized in all continents (van Kleunen et al., 2019) and is considered invasive and weedy in many regions (e.g., Firn et al., 2011). As such *P. pratensis* is also widespread in the Lower Lake Constance region and has recently increased in abundance in the more nutrient‐rich *Molinia* meadows around Lower Lake Constance (M. Peintinger, personal observation). Although Baumann's (1911) surveys had focused on habitat specialists, he reported the general presence of *P. pratensis* in meadows and along ditches, and in the *Molinia*‐meadow area of the Wollmatinger Ried (site 30 in Figure 1b).

As we could not collect *P. pratensis* seeds in the *Molinia* meadows, we ordered them from Rieger Hofmann GmbH and sowed them under the same conditions as described above for the target species. As the target plants (i.e., the individual seedlings of the target species) had to be planted over a 7‐week period (see below), we ensured that we would have enough seedlings of the competitor species *P. pratensis* of approximately the same developmental stage as the target species by sowing it on 9 and 17 May 2018. Seeds were sown on moistened potting substrate in polystyrol trays (32 × 50 × 6 cm^3^; MANNA Pikierschale, Romberg).

### 2.4 Experimental set‐up

To test how each of the 42 germinated *Molinia*‐meadow target species responded to nutrient addition and to competition with *P. pratensis*, and whether this response was related to occurrence frequency in 1911 and the change in occurrence frequency by 2017, we performed a common‐garden experiment in the Botanical Garden of the University of Konstanz (24 May to 28 September 2018). To reduce heat stress, the area was covered with a shading net (Nitsch & Sohn GmbH & Co KG), which reduced light intensity by 30%. Following a 2 × 2 factorial design, we grew each target species at high and low nutrient availability with and without competition (by eight *P. pratensis* plants). The experimental units were 3‐L circular plastic pots (Soparco 3‐L SMV; Le Musset) filled with about 1 kg of a 1:1 mixture of sand and fine vermiculite with 5% bentonite (Edasil^®^, Agrimont).

Due to non‐synchronous germination of the species, we had six different transplanting dates over a 7‐week period (from 22 May to 3 July 2018; Table S3). To allow the seedlings to acclimatize to the outside conditions, pots with seedlings were moved outside in a covered area 2 days prior to transplanting. Following transplanting of the target plants in the center of each pot (one target plant per pot), we assigned half of the pots to the competition treatment by planting eight similar‐sized seedlings of *P*. *pratensis* around the target plant, evenly spaced at c. 5 cm distance from the target plant (Table S4). Survival of the competitors was monitored weekly, and dead competitors were replaced until 3 weeks after the last transplanting event (i.e., until 25 July 2018); competitor mortality was 3% overall. Immediately after transplanting, the pots were placed back in the covered area outdoors for another 2 days, and then, they were moved to their final positions in the experiment.

To account for environmental heterogeneity in the garden, pots were organized in six blocks. In each block, we had ideally four plants per target species, one for each of the four competition‐by‐nutrient addition combinations (low nutrients without competition, high nutrients without competition, low nutrients with competition, high nutrients with competition). So, for species with sufficient seedlings, we had six replicates per treatment combination. However, for nine species, we did not have enough seedlings, resulting in 921 instead of 1,008 pots in total. The number of pots per block varied from 144 to 161 instead of 168 (see Tables S3 and S4 for details on the number of replicates). Within a block, each pot was randomly assigned to a position. To reduce herbivory by mollusks, we applied slug pellets (dosage c. 0.6 g/m^2^; Schneckenkorn Spiess‐Urania^®^ G2, Spiess‐Urania Chemicals GmbH) around the pots before the start of the experiment and following rainy weather. We watered all pots ad libitum to avoid severe drought stress. Nine days after transplanting, each pot received 60 ml of a 0.5‰ Universol Blue fertilizer solution (Scotts Universol^®^ blue 18‐11‐18‐2.5MgO + TE, Everis).

Thereafter, we started the low and high‐nutrient treatments by a weekly application of 60 ml Universol Blue solution (Scotts Universol^®^ blue 18‐11‐18‐2.5MgO + TE, Everis) per pot, respectively at 0.5‰ (“low,” corresponding to 5.4 mg N, 3.3 mg P and 5.4 mg K) or 1.0‰ (“high,” corresponding to 10.8 mg N, 6.6 mg P and 10.8 mg K). A pilot experiment of Liu and van Kleunen (2017) had shown that both the low‐ and high‐nutrient levels are still limiting in the sense that plants still achieved more growth at higher levels. We placed a dish below each pot (Sottovaso Siena Terracotta Plasticotto; 20 cm diameter) to prevent the loss of fertilizer solution that had drained from the bottom of each pot. Mortality of target plants was low (20 out of 921) and was more or less equally distributed among treatment combinations and species (Tables S3 and S4).

### 2.5 Measurements

To account for variation in initial sizes of the target plants, we measured the height of each target plant 6 days after transplanting. We ended the experiment between 24 and 28 September 2018, by harvesting the plants block by block. We separately collected the above‐ground biomass of the target plants and the collective above‐ground biomass of the competitor (*P*. *pratensis*). Additionally, to quantify below‐ground biomass, root‐mass fraction and total and specific root length, we collected the below‐ground biomass of competition‐free target plants. This was only done for pots without competition, because in pots with competition, target‐plant roots could not be separated from those of *P*. *pratensis*. We washed roots clean of substrate, stored them for 5–12 days in closed plastic bags filled with deionized water in a dark, cold room at c. 8°C until further processing. For plants with a thickened root or bulb, we separated these organs from the root system. Then, we selected a representative subsample of each root system and scanned it using Epson Scanners (11000 XL & 120000 XL, LA2400 Canada; settings: 8‐bit grayscale, reflective). We obtained root length for the subsample using the software WinRHIZO (v. 2017, Regents Instruments Inc.; Arsenault et al., 1995). All plant‐biomass samples were dried at 70°C for >72 hr before being weighed.

We calculated the specific root length of a plant as the ratio between the length and biomass of the root subsample. Based on the specific root length and the total root biomass of a plant, we estimated its total root length. We calculated the root‐mass fraction as the ratio between all below‐ground tissue (thickened root or bulb biomass + total root biomass) and the total biomass (above‐ground + below‐ground biomass).

### 2.6 Statistical analyses

To quantify the change in occurrence frequency for each of the 42 target species, we calculated an index of change. This index was calculated as a log‐response ratio: $\ln\left(\frac{N_{2017}+1}{N_{1911}+1}\right)$. Here, *N*_2017_ and *N*_1911_ correspond to the number of sites with species presence in 2017 and 1911, respectively. We added 1 to each number of sites to avoid zeros in the numerator of the log‐response ratio. We calculated the index based on the 14 sites that still had intact *Molinia* meadows in 2017. A negative index value indicates a decline in species' occurrence frequency, while a positive index value indicates an increase in species' occurrence frequency.

All analyses were conducted in R version 3.5.2 (R Core Team, 2018). To test how target‐plant traits responded to the competition and fertilizer treatments, and their interaction, and whether these responses were related to species occurrence frequency in 1911 and to the index of change in occurrence frequency, we fitted linear mixed models (LMM) using the “lme” function of the *nlme* package (Pinheiro et al., 2018). As fixed factors, we included Competition treatment (with vs. without *P. pratensis*) and Fertilizer treatment (low‐ vs. high‐nutrient levels) and their interaction. Competition treatment was not included in the models for root traits, as those were only measured in the competition‐free pots. Furthermore, we included the covariates Occurrence frequency in 1911 (centered) and the Index of change in occurrence frequency (centered), and all possible interactions with Competition treatment and Fertilizer treatment.

To account for variation in initial size of the seedlings and for variation in the duration of the experiment (83–125 days due to different starting dates), we included Initial plant height (log‐transformed, centered) and the Growing time (centered) as covariates. To account for non‐independence of plants of the same species and taxonomic relatedness among species, we included target species identity nested in plant family as random factors. Initially, we had also included block as a random factor, but as some models had convergence problems and block did not explain much variation, we removed it from all models. To improve normality of the residuals, the target above‐ground biomass, total root length and specific root length were natural log (ln) transformed, and target below‐ground biomass and the ratio of target above‐ground to total above‐ground biomass were log_10_‐transformed. To account for heterogeneity of variance among target species, we allowed each target species to have its own variance (Table S5) by applying the “varIdent” function of the *nlme* package (Pinheiro et al., 2018).

Significances of the fixed terms in the models were assessed with log‐likelihood ratio tests (LRTs) by constructing models (with the maximum‐likelihood [ML] method) that excluded one by one the fixed terms of interest and comparing these models to their respective reference models (Zuur et al., 2009). The detailed scheme of the order in which terms were removed are shown in Tables S6 and S7.

## 3 RESULTS

### 3.1 Effects of competition and nutrient addition on above‐ground biomass

**FIGURE 2:**
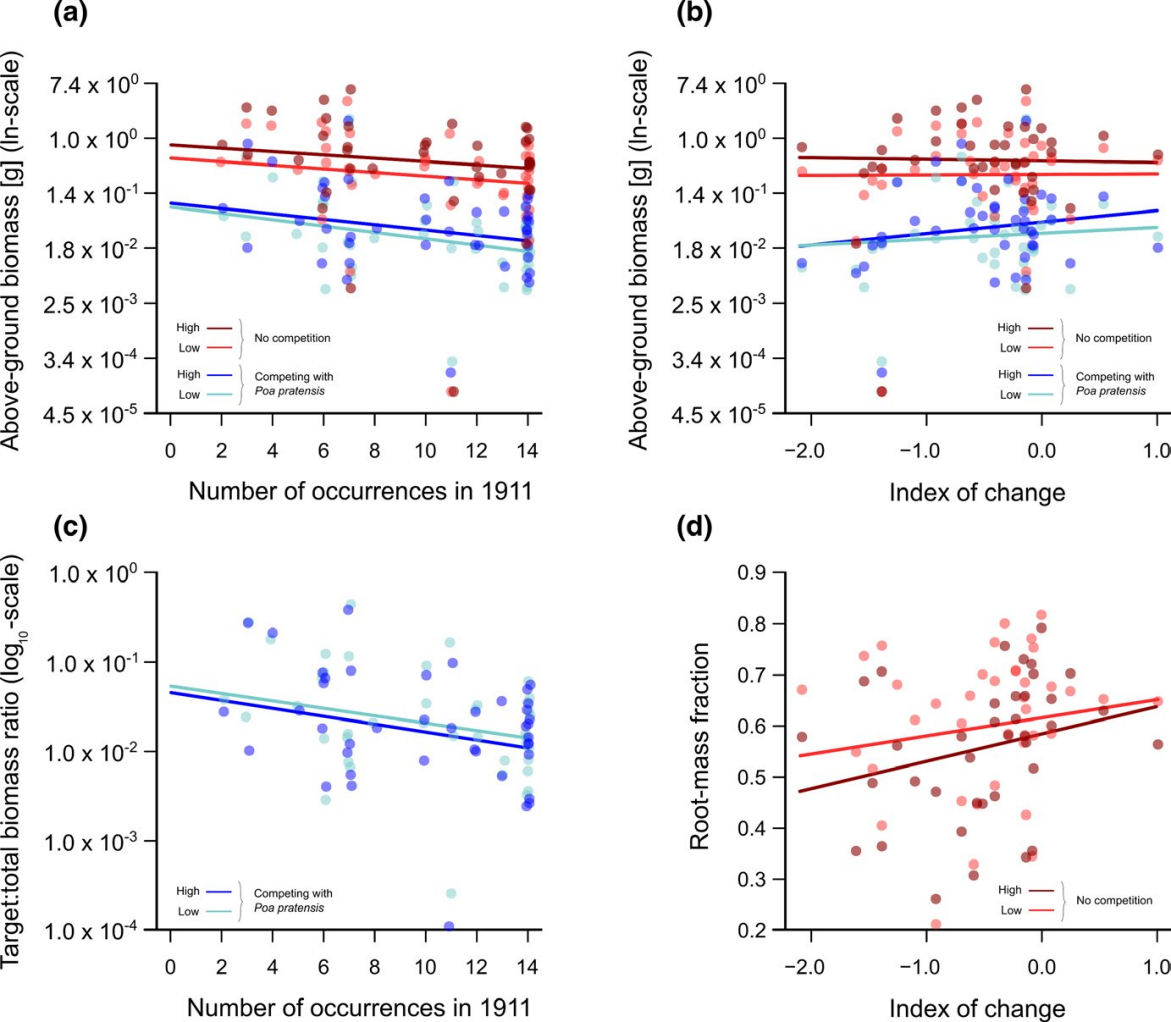
(a) Relationships between above‐ground target biomass and the number of sites with occurrences in 1911 for plants in all four nutrient‐addition and competition treatments. (b) Relationships between above‐ground target biomass and the index of change in occurrence frequency for plants in all four nutrient‐addition and competition treatments. (c) Relationships between the ratio of target above‐ground biomass to total above‐ground biomass and the number of sites with occurrences in 1911 for plants in the low and high nutrient‐addition treatments. (d) Relationships between the root‐mass fraction and the index of change in occurrence frequency for plants in the low and high nutrient‐addition treatments. Points indicate the mean values per species for each treatment combination (note that a small horizontal jitter was applied). Lines are the modeled responses based on marginal means (i.e., using the model estimates shown in Tables S8 and S9)

Across all 42 study species of the pot experiment, nutrient addition had a significant positive effect on above‐ground biomass production, and competition with *P. pratensis* had a significant negative effect (Figure 2a; Figure S1a; Table 1). The effect of nutrient addition, however, was generally small. The effect of competition was significantly stronger in the high‐nutrient treatment than in the low‐nutrient treatment (significant Fertilizer × Competition interaction in Table 1).

**TABLE 1:** Results of linear mixed model analyses of the effect of nutrient addition and competition with *Poa pratensis* on above‐ground target biomass, and the ratio of above‐ground target biomass to combined above‐ground biomass of target and competitor (*P. pratensis*)

Fixed effects	ln (above‐ground biomass)			log_10_ (target: total biomass ratio)		
	LRT^c^		*p*‐value	LRT^c^		*p*‐value
Covariable: Initial plant height	12.08		**<0.001**	24.97		**<0.001**
Covariable: Growing time	1.78		0.182	‐		‐
1911 Occurrence frequency	4.39		**0.036**	6.01		**0.014**
Index of change in occurrence frequency^a^	0.19		0.661	0.50		0.478
Competition (without vs. with *Poa pratensis*)	1,069.21		**<0.001**	‐		‐
Fertilizer (low‐nutrient vs. high‐nutrient)	76.96		**<0.001**	17.63		**<0.001**
Fertilizer: Competition	8.87		**0.003**	‐		‐
Competition: 1911	11.03		**<0.001**	‐		‐
Fertilizer: 1911	1.20		0.273	0.03		0.860
Competition: Index of change in occurrence frequency	8.55		**0.003**	‐		‐
Fertilizer: Index of change in occurrence frequency	0.03		0.863	2.93		0.087
Fertilizer: Competition: 1911	0.61		0.433	‐		‐
Fertilizer: Competition: Index of change in occurrence frequency	1.52		0.218	‐		‐
**Random effects**	**Levels**	***SD***	**Residual**	**Levels**	***SD***	**Residual**
Family	22	0.8082		22	0.3388	
Species within family^b^	42	0.8714	0.8721	42	0.3676	0.3909
Number of observations	877			442		

Note

The significance of the fixed terms was assessed using likelihood‐ratio tests (LRT) based on models with and without the term of interest (see Tables S5 and S6 for details). Estimates from the full models are given in Table S8.

^a^ Index of change in occurrence frequency was calculated as the log‐response ratio of the number of sites in which a species was present in 2017 relative to 1911.

^b^ Standard deviations for the individual species' random terms of the full model are shown in Table S5.

^c^ All likelihood‐ratio tests had one degree of freedom.

There was a negative relationship between occurrence frequency in 1911 and above‐ground biomass production in the experiment (Figure 2a; Table 1). This negative relationship was strongest in the presence of competition, indicating that species that were widespread in 1911 suffered the most from competition in our experiment (Figure 2a; significant Competition × 1911 interaction in Table 1). In the absence of competition, above‐ground biomass production was unrelated to the change in species occurrence frequency between 1911 and 2017. In the presence of competition, species that had declined more were those with less biomass in the experiment (Figure 2b; significant Competition × Index of change interaction in Table 1).

In competition with *P. pratensis*, the proportion of target relative to total above‐ground biomass per pot (i.e., the target:total above‐ground biomass ratio) was slightly, but significantly smaller in the high‐nutrient treatment than in the low‐nutrient treatment (Figure 2c; Figure S1b; Table 1). This ratio significantly decreased with increasing occurrence frequency in 1911, and this did not depend on the nutrient treatment (Figure 2c; Table 1). The target:total biomass ratio, however, was not significantly related to the index of change in occurrence frequency of the species (Figure S1b; Table 1).

### 3.2 Effects of nutrient addition on below‐ground traits

Below‐ground traits were only available for the target plants grown without competition. The high‐nutrient treatment resulted in an absolute increase of below‐ground biomass and total root length, and a decrease of the root‐mass fraction, but had no significant effect on specific root length (Figure S2; Table 2). The negative effect of the high‐nutrient treatment on root‐mass fraction decreased with the index of change in occurrence frequency of the species (Figure 2d; significant Fertilizer × Index of change interaction in Table 2).

**TABLE 2:** Results of linear mixed model analyses of below‐ground target biomass and root traits when grown without competition

Fixed effects	log_10_ (below‐ground biomass)			Root‐mass fraction			ln (total root length)			ln (specific root length)		
	LRT^c^		*p*‐value	LRT^c^		*p*‐value	LRT^c^		*p*‐value	LRT^c^		*p*‐value
Initial plant height	30.26		**<0.001**	4.87		**0.027**	12.61		**<0.001**	‐		‐
Growing time	11.24		**<0.001**	2.62		0.106	8.33		**0.004**	2.29		0.131
1911 Occurrence frequency	0.29		0.592	3.03		0.082	0.70		0.403	0.42		0.516
Index of change in occurrence frequency^a^	0.21		0.649	1.77		0.183	<0.005		0.958	1.16		0.281
Fertilizer (low‐nutrient vs. high‐nutrient)	71.56		**<0.001**	48.85		**<0.001**	29.39		**<0.001**	0.07		0.797
Fertilizer: 1911	0.00		0.952	1.47		0.226	2.46		0.117	1.37		0.242
Fertilizer: Index of change in occurrence frequency	0.01		0.922	6.19		**0.013**	0.01		0.909	1.77		0.183
**Random effects**	**Levels**	***SD***	**Residual**	**Levels**	***SD***	**Residual**	**Levels**	***SD***	**Residual**	**Levels**	***SD***	**Residual**
Family	20	0.2587		20	0.0255		20	0.5921		20	0.2054	
Species within family^b^	39	0.5315	0.2835	39	0.1206	0.0803	39	1.4091	0.5621	39	0.5448	0.2431
Number of observations	434			433			434			434		

Note

The significance of the fixed terms was assessed using likelihood‐ratio tests (LRT) based on models with and without the term of interest (see Table S7 for details). Estimates from the full models are given in Table S9.

^a^ Index of change in occurrence frequency was calculated as the log‐response ratio of the number of sites in which a species was present in 2017 relative to 1911.

^b^ Standard deviations for the individual species' random terms of the full model are shown in Table S5.

^c^ All likelihood‐ratio tests had one degree of freedom.

## 4 DISCUSSION

Focusing on 42 characteristic *Molinia*‐meadow species, our study is one of the first to test whether historical occurrence frequencies and recent changes are related to sensitivities of the species to environmental change. Our experiment, which aimed to disentangle the influence of two potentially contributing factors, revealed species‐specific responses to nutrient addition and competition with the grass *P. pratensis*. As expected, target plants generally produced more biomass when grown without competition and when they received more nutrients, even though the effect of nutrient addition alone was relatively small. Nutrient addition, however, slightly reduced the proportional biomass of the target plants and thus amplified the negative effect of competition. This suggests that the competitor grass, *P. pratensis*, capitalized more on the additional nutrients than the *Molinia*‐meadow habitat specialists. This is in line with the results of a recent global meta‐analysis showing that grasses on average capitalize more on nitrogen addition than forbs do (You et al., 2017). We furthermore found that the more widespread a species was in 1911, the less biomass it produced in our experiment, and the stronger it was affected by competition. Similarly, the stronger a species had declined since 1911, the stronger it was affected by competition. Our results thus suggest that increased nutrient inputs in *Molinia* meadows may have increased the competitive strength of grasses like *P. pratensis*. Although this species was not very abundant in *Molinia* meadows in 1911 (Baumann, 1911), it has become more abundant there recently (M. Peintinger, personal observation). Subsequently, variation in the responses to increased competition may explain why some characteristic *Molinia*‐meadow species have declined more strongly in their occurrence frequency than others.

### 4.1 Responses to nutrient addition and competition

Baumann (1911) had already expressed concerns that land‐use conversion would reduce the future size and quality of lakeshore habitats. Our results, however, indicate that the overall decrease in the occurrence frequency of *Molinia*‐meadow species is not solely due to habitat destruction but also occurs in the remaining intact *Molinia* meadows. Local extinctions have occurred despite serious efforts to preserve the remaining *Molinia* meadows. The exact reasons for this are unknown, but our experiment provides evidence that, like in many other naturally nutrient‐poor habitats, and it is likely that increased nutrient loads might have contributed to the observed species declines. Other studies also provide evidence for this mechanism. For example, experimental nutrient addition for 15 years reduced species richness in a Czech *Molinia* meadow, and this could be countered by removing *Molinia caerulea* (Lepš, 2014), which is also one of the dominant grasses in the *Molinia* meadows around Lower Lake Constance. Similarly, in Dutch heathlands, atmospheric nitrogen deposition coincided with an increasing dominance of *M. caerulea* (Aerts & Berendse, 1988; Aerts et al., 1990; Aerts & Bobbink, 1999). Furthermore, it was shown that *M. caerulea* could only outcompete the heather *Erica tetralix* when the plants were grown under high‐nutrient conditions (Aerts & Berendse, 1988; Aerts et al., 1990; Aerts & Bobbink, 1999). Similarly, our experiment showed that the effect of competition with the grass *P. pratensis* was strongest when the *Molinia*‐meadow plants were grown under high‐nutrient conditions. The slight but significant decrease in target:total biomass ratio in response to increased nutrients in our experiment further supports the idea that grasses like *P. pratensis* and *M. caerulea* have become more prevalent in *Molinia* meadows as a consequence of increased nutrient loads that resulted in increased competitive strength.

### 4.2 Drivers of variation in historical occurrence frequencies

We found that *Molinia*‐meadow species with high occurrence frequencies in 1911 on average produced less biomass than the ones with low occurrence frequencies. This indicates that, one century ago, small‐statured species were more successful in the *Molinia* meadows than tall species. This probably reflects that the meadows were then still nutrient‐poor, favoring smaller species with a more conservative resource use. The species that were more widespread in 1911 also suffered more strongly from competition with *P. pratensis*. This is most likely due to their relatively small size, which makes them more prone to be shaded by the faster growing grass. Indeed, it has been shown that the outcome of competition is at least partly determined by differences in height and intrinsic growth rates between species (Dostàl, 2011; Zhang & van Kleunen, 2019).

### 4.3 Drivers of changes in occurrence frequencies

Our findings underscore the role of competition in contributing to changes in the occurrence frequencies of *Molina*‐meadow habitat specialists. Species that had a stable or increased occurrence frequency suffered less from competition with the acquisitive generalist grass *P. pratensis* than the ones that decreased in occurrence frequency. Similarly, based on a regional comparison of changes in species occurrences among the UK and Estonia, plant species that were less resistant to anthropogenic activities were the ones that declined the most over a 30‐year period (Laanisto et al., 2015). Furthermore, in a study of calcareous nutrient‐poor grasslands in the Swiss Jura Mountains, Fischer and Stöcklin (1997) found that many specialist species characteristic for that habitat type had been replaced by common generalist species (including *P. pratensis*) over a 35‐year period. In line with these other studies, our habitat‐specific study in *Molinia* meadows also suggests that habitat specialists are the ones that suffer the most from human‐caused environmental changes.

Root morphology and the relative allocation of biomass to roots likely affect how plants respond to changes in nutrient availability. As plants generally grow larger when nutrient availability increases, it is not surprising that below‐ground biomass and total root length of the *Molinia*‐meadow species were also largest in the high‐nutrient treatment. The specific root length, which indicates whether plants produce thinner or thicker roots (Bergmann et al., 2020; and respectively associated with high nutrient acquisition and reliance on mycorrhizal fungi for resource acquisition; Ma et al., 2018; Valverde‐Barrantes et al., 2017), was not affected by the nutrient treatment. However, in line with predictions of the functional equilibrium theory (Brouwer, 1962; also see Bloom et al., 1985) and many experimental studies (Freschet et al., 2015; Liu & van Kleunen, 2017; Poorter & Nagel, 2000), we found that plants allocated relatively less biomass to roots (i.e., decreased their root‐mass fraction) in the high‐nutrient treatment. Interestingly, however, the species that had a stable or increased occurrence frequency were the least plastic in their response of root‐mass fraction to nutrient addition. Under high‐nutrient conditions, these species actually had a higher root‐mass fraction than the less stable species. Possibly, this unexpected finding reflects that the stable species store more resources in their root systems and are therefore more likely to persist in the long term. Storage of resources might be particularly relevant for persistence in the *Molinia* meadows given that they do not only experience annual mowing but also have to endure long flooding events, at least once every 10–20 years (LUBW, 2011). The positive relationship between root‐mass fraction and our index of change can also suggest that below‐ground competition might be an important driver for the change in occurrence frequency, as was recently shown for the impacts of invasive species on native species (Broadbent et al., 2018). The duration of our study, however, might have been too short to fully capture the effects of below‐ground competition.

### 4.4 Some limitations of our study

Although our large multi‐species experiment included almost half of the 91 *Molinia*‐meadow habitat specialists and 22 families, some taxonomic groups could not be included. In particular, we could not include any of the 14 *Molinia*‐meadow representatives of the Orchidaceae, because these are notoriously difficult to germinate. It is not clear how this taxonomic bias may have affected our results. Additionally, as we had to limit the number of experimental treatments, we cannot exclude that other factors (e.g., on‐site changes in water‐level‐fluctuation patterns and changes in management practices) contributed to the observed variation in occurrence frequency of *Molinia* meadow species. Furthermore, it would have been interesting to test how the species would respond to nutrient addition and competition in the presence of their natural soil biota. Plant‐soil feedbacks can have important consequences for community dynamics (Kardol et al., 2013). For example, Klironomos (2002), showed that locally rare species suffered from strong negative plant‐soil feedbacks, while invasive alien species showed positive plant soil feedbacks. Furthermore, our competition treatment was limited to a single grass species, and it thus remains to be tested whether other competitors would have had similar effects. Finally, it is debatable whether biomass produced in a single year provides a good proxy for the lifetime performance of perennials. Common‐garden‐ and greenhouse‐based experimental setups like ours also ignore demographic aspects such as recruitment and mortality (see Goldberg et al., 1999), which likely are important in natural grasslands, especially those where disturbance is an important factor.

## 5 CONCLUSIONS

Our outdoor pot experiment with 42 *Molinia*‐meadow specialist species indicates that the common generalist grass *P. pratensis* takes more advantage of additional nutrients and can competitively suppress most of the characteristic *Molinia*‐meadow species. Indeed, species that were most affected by competition in the experiment were those that have declined most in their occurrence frequency. Therefore, we conclude that it is likely that increased nutrient loads in intact *Molinia*‐meadow remnants have benefited grass species that do well under high nutrient‐availability, and, in addition to other factors, contributed to the local extinction of *Molinia*‐meadow species. This highlights that potential increase of nutrient loads in this ecosystem (e.g., through atmospheric nitrogen deposition) should be considered in management practices aiming to prevent further habitat degradation.

## CONFLICT OF INTEREST

The authors have no conflict of interest to declare.

## AUTHOR CONTRIBUTIONS

**Stefanie Höckendorff:** Data curation (equal); Formal analysis (equal); Investigation (lead); Methodology (equal); Visualization (lead); Writing‐original draft (lead); Writing‐review & editing (equal). **Markus Peintinger:** Conceptualization (supporting); Data curation (equal); Resources (lead); Writing‐review & editing (supporting). **Felicitas Fiedler:** Investigation (supporting); Methodology (supporting). **Marc Stift:** Formal analysis (equal); Methodology (supporting); Visualization (lead); Writing‐original draft (equal); Writing‐review & editing (equal). **Mark van Kleunen:** Conceptualization (lead); Formal analysis (equal); Funding acquisition (lead); Methodology (equal); Resources (equal); Supervision (lead); Visualization (supporting); Writing‐original draft (equal); Writing‐review & editing (lead).

## Supporting information

Figure S1

Figure S2

Table S1‐S9

Supplementary Material

## Data Availability

The data used in this manuscript were deposited on Dryad: https://doi.org/10.5061/dryad.qbzkh18gk.
