# Supplementary material for: Declines in occurrence of plants characteristic for a nutrient‐poor meadow habitat are partly explained by their responses to nutrient addition and competition
Source: Ecol Evol. 2021 Mar 7;11(9):4058–70. doi: 10.1002/ece3.7306 (PMC8093689; doi:10.1002/ece3.7306)
Supplement: Supplementary file 3 — Table S1‐S9 [file ECE3-11-4058-s004.docx]

**Appendix Table S1.** The 2017-classification of the 37 sites around Lower Lake Constance which contained *Molinia* meadows in 1911 according to Baumann (1911). Classifications followed the plant sociological alliances according to Braun-Blanquet system (Oberdorfer 2001) and biotope classifications of the State Baden Württemberg (LUBW, 2020). Fourteen sites had a vegetation that could be classified as Molinion caeruleae and Primulo-Schoenetum and thus are considered to be intact *Molinia* meadows and were considered in this study. Six sites had a vegetation that could be classified as tall herb communities (Filipendulion), tall sedge communities (Magnocaricion) or nutrient-rich wet meadows (Calthion) and were thus considered to be severely degraded wetland remnants of the original *Molinia*-meadow habitat. In the 17 remaining sites the original *Molinia*-meadow habitat had been destroyed (i.e. other types of grassland or construction on top). Topographic mapping is according to “1:25.000 des Landesvermessungsamtes Baden-Württemberg”, the Q-number indicates the quadrant direction 1: NW, 2: NE, 3: SW, 4: SE. Area estimates are as follows: destroyed=0 [0 ha]; degraded=NA [NA] and three options for intact meadows. For sites in Switzerland, the area size estimates were based on aerial photographs from //map.geo.admin.ch. For sites in Germany, the additional number in brackets was obtained from information sheets of the Biotopkartierung Baden-Württemberg LUBW https://udo.lubw.baden-wuerttemberg.de.

| **Locations with Molinia meadows in 1911** | **No on map** | **Country** | ***Molinia* meadow classification 2017** | **Topographic mapping**  **(MTB/Q.)** | **Estimated area in hectares^a^** |
| --- | --- | --- | --- | --- | --- |
| Tägermoos^b^, Ziegelhütte | 1 | Switzerland | destroyed | 8320/2 | 0 |
| Gottlieben | 2 | Switzerland | still intact | 8320/2 | 1-10 |
| Triboltingen | 3 | Switzerland | still intact | 8320/2 | 1-10 |
| Ermatingen^c^ – Agerstenbach | 4 | Switzerland | still intact | 8320/2 | <1 |
| Ermatingen^c^ – Bucher, Westerfeld, Böschen | 5 | Switzerland | destroyed | 8320/2 | 0 |
| Mannenbach | 6 | Switzerland | destroyed | 8320/1 | 0 |
| Steckborn | 7 | Switzerland | destroyed | 8319/2 | 0 |
| Glarisegg | 8 | Switzerland | destroyed | 8319/2 | 0 |
| Mammern | 9 | Switzerland | destroyed | 8319/3 | 0 |
| Eschenz | 10 | Switzerland | severely degraded | 8319/1 | NA^d^ |
| Stein am Rhein | 11 | Switzerland | destroyed | 8319/1 | 0 |
| Öhningen Stiegen | 12 | Germany | destroyed | 8319/1 | 0 |
| Öhningen Oberstaad | 13 | Germany | severely degraded | 8319/1 | NA |
| Kattenhorn | 14 | Germany | destroyed | 8319/1 | 0 |
| Wangen^e^ | 15 | Germany | still intact | 8319/2 | <1 (0.27) |
| Schloss Marbach^e^ | 16 | Germany | destroyed | 8319/2 | 0 |
| Hemmenhofen | 17 | Germany | severely degraded | 8319/2 | NA |
| Gaienhofen | 18 | Germany | still intact | 8319/2 | 1-10 (3.6) |
| Horn, Hornstad | 19 | Germany | still intact | 8320/1 | 1-10 (2.3) |
| Gundholzen | 20 | Germany | severely degraded | 8219/4 | NA |
| Iznang | 21 | Germany | destroyed | 8219/4 | 0 |
| Moos | 22 | Germany | still intact | 8219/4 | <1 (0.24) |
| Radolfzell – Lake reed near Moos | 23 | Germany | still intact | 8219/4 | >10 (37) |
| Radolfzell - Mettnau | 24 | Germany | still intact | 8219/4, 8220/3 | >10 (16) |
| Radolfzell – Lake reed near Markelfingen | 25 | Germany | still intact | 8220/3 | 1-10 (4.8) |
| Markelfingen | 26 | Germany | still intact | 8220/3 | 1-10 (1.3) |
| Allensbach | 27 | Germany | destroyed | 8220/3 | 0 |
| Hegne | 28 | Germany | severely degraded | 8220/4 | NA |
| Giehrenmoos | 29 | Germany | still intact | 8220/4, 8320/2 | >10 (74)^f^ |
| Wollmatinger Ried | 30 | Germany | still intact | 8320/2 |  |
| Reichenau – Schopflen, Streichen, Fehrenhorn, Bibershorn, Mauershorn | 31 | Germany | severely degraded | 8320/2 | NA |
| Reichenau – Bauershorn, Mittelzell | 32 | Germany | destroyed | 8220/3 | 0 |
| Reichenauer – Steinener Weg | 33 | Germany | still intact | 8220/3 | <1 (0.57) |
| Reichenau – Unterzell, Bürglehorn, Lake reeds near Unterzell | 34 | Germany | destroyed | 8220/3 | 0 |
| Reichenau – Bradlen, Melchershorn | 35 | Germany | destroyed | 8320/1 | 0 |
| Konstanz Seerhein^g^ – Stromeyersdorf, Bleiche | 36 | Germany | destroyed | 8320/2 | 0 |
| Konstanz Seerhein^g^ – Rheingut, Paradies | 37 | Germany | destroyed | 8320/2 | 0 |
| ^a^ Size Estimates for Germany were obtained from LUBW, estimates for Switzerland from aerial pictures on //map.geo.admin.ch. Numbers  in brackets refer to the exact site hectares obtained from LUBW. This was not possible for sites located in Switzerland.  ^b^ was also included in Konstanz Seerhein in Peintinger (2012), extracted from Baumann (1911)  ^c^ listed as a single site in Peintinger (2012)  ^d^ NA as estimate could not be obtained from LUBW.  ^e^ listed as a single site in Peintinger (2012)  ^f^ Giehrenmoos and Wollmatinger Ried each are bigger than 10 hectares, however separate sizes could not be obtained. 74 indicates total area of both.  ^g^ listed as a single site in Peintinger (2012) | | | | | |

**Appendix Table S2** The 1911 vs. 2017 Molinia-meadow site occurrence frequencies for the 54 selected target species (42 used in our experiment, and 12 that could not be used, because they did not germinate in sufficient numbers). Seed families of the target species were sown in multiple pots (Sown seed families) on one of two different sowing dates; each seed family was sown in a separate pot.

| **Species (perennial unless indicated otherwise)** | **Family** | **Number of sites in which a species was found to be present** | | | | | | **Index of change in occurrence frequency^c^** | **Origin of material^d^** | **Sown**  **seed families** | **Date sown (2018)** |
| --- | --- | --- | --- | --- | --- | --- | --- | --- | --- | --- | --- |
|  |  | **14 intact  meadows^a^** | | **6 degraded meadows^a^** | | **17 destroyed meadows^a^** | |  |  |  |  |
|  |  | **1911** | **2017** | **1911** | **2017** | **1911** | **2017** |  |  |  |  |
| **42 species included in our experiment** | | | | | | | | | | | |
| *Agrimonia eupatoria* | Rosaceae | 2 | 1 | 2 | 0 | 1 | 0 | -0.405 | GM | 10 | 04/30 |
| *Allium angulosum* | Alliaceae | 14 | 9 | 3 | 1 | 7 | 0 | -0.405 | GM, RAR | 12 | 05/07 |
| *Allium schoenoprasum* | Alliaceae | *14^b^* | 11 | *6^b^* | 1 | *13^b^* | 0 | -0.223 | WM | 12 | 05/07 |
| *Allium suaveolens* | Alliaceae | 6 | 8 | 3 | 1 | 2 | 0 | 0.251 | GM, WM | 12 | 05/07 |
| *Aquilegia atrata* | Ranunculaceae | 5 | 1 | 3 | 0 | 3 | 0 | -1.099 | MS | 12 | 04/30 |
| *Betonica officinalis* | Lamiaceae | *14^b^* | 5 | *6^b^* | 1 | *13^b^* | 0 | -0.916 | WM | 13 | 05/07 |
| *Carex davalliana* | Cyperaceae | 12 | 2 | 6 | 0 | 13 | 0 | -1.466 | MT | 20 | 05/07 |
| *Carex distans* | Cyperaceae | 11 | 5 | 6 | 1 | 7 | 0 | -0.693 | GM | 12 | 05/07 |
| *Carex flava* | Cyperaceae | 10 | 11 | 5 | 2 | 8 | 0 | 0.087 | H | 12 | 05/07 |
| *Carex hostiana* | Cyperaceae | 13 | 11 | 5 | 0 | 8 | 0 | -0.154 | MT | 15 | 05/07 |
| *Centaurium erythraeae* | Gentianaceae | 7 | 1 | 3 | 0 | 6 | 0 | -1.386 | GM | 12 | 04/30 |
| *Cirsium tuberosum* | Asteraceae | 6 | 1 | 1 | 0 | 1 | 0 | -1.253 | G*, J* | 2 | 05/07 |
| *Dianthus carthusianorum* | Caryophyllaceae | 3 | 1 | 3 | 0 | 2 | 0 | -0.693 | GM | 11 | 04/30 |
| *Eriophorum latifolium* | Cyperaceae | *14^b^* | 2 | *6^b^* | 0 | *13^b^* | 0 | -1.609 | MT | 12 | 05/07 |
| *Galium boreale* | Rubiaceae | 12 | 6 | 6 | 1 | 7 | 0 | -0.619 | GM | 11 | 05/07 |
| *Gentiana pneumonanthe* | Gentianaceae | 14 | 12 | 5 | 1 | 9 | 0 | -0.143 | GM | 12 | 04/30 |
| *Gratiola officinalis* | Plantaginaceae | 13 | 2 | 5 | 0 | 10 | 0 | -1.540 | H, K* | 12 | 05/07 |
| *Inula salicina* | Asteraceae | 10 | 11 | 4 | 0 | 5 | 0 | 0.087 | GM, RAR | 12 | 05/07 |
| *Iris sibirica* | Iridaceae | 11 | 10 | 4 | 2 | 3 | 0 | -0.087 | GM | 12 | 04/30 |
| *Juncus alpinus* | Juncaceae | 6 | 11 | 5 | 4 | 8 | 0 | 0.539 | H | 12 | 05/07 |
| *Juncus subnodulosus* | Juncaceae | *14^b^* | 12 | *6^b^* | 4 | *13^b^* | 0 | -0.143 | GM | 12 | 05/07 |
| *Linum catharticum* (annual) | Linaceae | *14^b^* | 9 | *6^b^* | 0 | *13^b^* | 0 | -0.405 | GM | 17 | 05/07 |
| *Lotus maritimus* | Fabaceae | 7 | 5 | 3 | 0 | 6 | 0 | -0.288 | MS | 15 | 04/30 |
| *Lotus pedunculatus* | Fabaceae | 6 | 3 | 3 | 0 | 2 | 0 | -0.560 | H | 12 | 05/07 |
| *Lysimachia vulgaris* | Primulaceae | *14^b^* | 14 | *6^b^* | 4 | *13^b^* | 1 | 0 | GM | 12 | 04/30 |
| *Menyanthes trifoliata* | Menyanthaceae | 7 | 0 | 5 | 0 | 4 | 0 | -2.079 | BR*, O* | 6 | 04/30 |
| *Molinia caerulea* | Poaceae | *14^b^* | 13 | *6^b^* | 5 | *13^b^* | 0 | -0.069 | WM | 12 | 05/07 |
| *Ononis spinosa* | Fabaceae | 7 | 6 | 3 | 0 | 2 | 0 | -0.134 | GM | 12 | 05/07 |
| *Polygala amarella* | Polygalaceae | 7 | 6 | 3 | 0 | 5 | 0 | -0.134 | SAE, MF | 12 | 05/07 |
| *Potentilla erecta* | Rosaceae | *14^b^* | 13 | *6^b^* | 2 | *13^b^* | 1 | -0.069 | K* | 19 | 04/30 |
| *Primula farinosa* | Primulaceae | 8 | 4 | 4 | 0 | 7 | 0 | -0.588 | WM | 12 | 04/30 |
| *Ranunculus flammula* | Ranunculaceae | *14^b^* | 8 | *6^b^* | 0 | *13^b^* | 0 | -0.511 | WM | 12 | 04/30 |
| *Ranunculus nemorosus* | Ranunculaceae | 3 | 10 | 3 | 2 | 2 | 0 | 1.012 | GM | 14 | 04/30 |
| *Sanguisorba officinalis* | Rosaceae | *14^b^* | 12 | *6^b^* | 5 | *13^b^* | 1 | -0.143 | RAR | 17 | 04/30 |
| *Schoenus nigricans* | Cyperaceae | 12 | 11 | 6 | 1 | 10 | 0 | -0.080 | MT | 14 | 05/07 |
| *Serratula tinctoria* | Asteraceae | *14^b^* | 11 | *6^b^* | 0 | *13^b^* | 0 | -0.223 | RAR | 13 | 05/07 |
| *Succisa pratensis* | Caprifoliaceae | *14^b^* | 9 | *6^b^* | 1 | *13^b^* | 0 | -0.405 | GM | 15 | 04/30 |
| *Taraxacum sect. Palustria* | Asteraceae | 10 | 7 | 4 | 0 | 6 | 0 | -0.318 | GM | 11 | 05/07 |
| *Tofieldia calyculata* | Tofieldiaceae | 11 | 2 | 3 | 0 | 9 | 0 | -1.386 | S* | 3 | 04/30 |
| *Triglochin palustre* | Juncaginaceae | 7 | 1 | 3 | 0 | 5 | 0 | -1.386 | MS, R | 12 | 05/07 |
| *Veronica scutellata* | Plantaginaceae | 4 | 1 | 1 | 0 | 1 | 0 | -0.916 | N*, RAR | 12 | 05/07 |
| *Vincetoxicum hirundinaria* | Apocynaceae | 6 | 5 | 2 | 2 | 1 | 0 | -0.154 | B* | 8 | 05/07 |
|  |  |  |  |  |  |  |  |  |  |  |  |
| **12 species initially selected, but not included in experiment (due to lack of seeds or problems with germination)** | | | | | | | | | | | |
| *Blysmus compressus* | Cyperaceae | 7 | 0 | 2 | 0 | 3 | 0 | -2.079 | MS | 12 | 05/07 |
| *Carex flacca* | Cyperaceae | *14^a^* | 12 | *6^b^* | 2 | *13^b^* | 1 | -0.143 | H | 12 | 05/07 |
| *Carex panicea* | Cyperaceae | *14^b^* | 13 | *6^b^* | 3 | *13^b^* | 0 | -0.069 | GM | 20 | 04/30 |
| *Carex tomentosa* | Cyperaceae | 11 | 11 | 3 | 1 | 11 | 0 | 0 | GM,WM | 13 | 05/07 |
| *Cladium mariscus* | Cyperaceae | 5 | 11 | 3 | 2 | 0 | 0 | 0.693 | H | 12 | 05/07 |
| *Drosera longifolia* | Droseraceae | 2 | 1 | 3 | 0 | 7 | 0 | -0.405 | W | 12 | 04/30 |
| *Eleocharis uniglumis* | Cyperaceae | 6 | 11 | 2 | 1 | 4 | 0 | 0.539 | MT | 16 | 04/30 |
| *Epipactis palustris* | Orchidaceae | 12 | 7 | 6 | 1 | 8 | 0 | -0.486 | BR*, WM | 11 | 04/30 |
| *Parnassia palustris* | Celastraceae | *14^b^* | 7 | *6^b^* | 0 | *13^b^* | 0 | -0.629 | GM | 12 | 04/30 |
| *Pinguicula vulgaris* | Lentibulariaceae | 7 | 3 | 4 | 0 | 7 | 0 | -0.693 | W | 12 | 05/07 |
| *Selinum carvifolia* | Apiaceae | 6 | 7 | 2 | 0 | 3 | 0 | 0.134 | WM | 12 | 05/07 |
| *Viola elatior* | Violaceae | 0 | 1 | 0 | 0 | 0 | 0 | 0.693 | RAR | 7 | 05/07 |
| ^a^ This classification is based on the situation in 2017. Back in 1911, all 37 sites had been classified as *Molinia* meadows by Baumann (1911). For more detailed information on the sites, see Appendix Table S1.  ^b^ These species were listed as “common” by Baumann (1911), which implies that they were present in all sites. However, to be conservative,  we assigned to them the highest number of occurrences reported for any of the other species.  ^c^ For the 14 *Molinia* meadows still intact in 2017, the Index of change in occurrence frequency was calculated as the log-response ratio of the  number of sites in which a species was present in 2017 relative to 1911  ^d^ Abbreviations with an * indicate material sourced from botanical gardens. Abbreviations: B= Botanical Garden Braunschweig, BR= Botanical Garden Bormio Rezia, G= Botanical Garden Gieβen, GM= Giehrenmoos, H= Horn, J= Botanical Garden Jibou, K= Konstanz, MF= Markelfingen, MS= Mindelsee, MT= Mettnau, N=Botanical Garden Nantes, O= Botanical Garden Oslo, R= Botanical Garden Regensburg, RAR= Radolfzeller Aachried, S= Botanical Garden Salzburg, SAE= Schrännen, W= Weiler, WM= Wollmatinger Ried. | | | | | | | | | | | |

**Appendix Table S3.** Distribution of the 42 individual target species over the six experimental blocks. Cases with mortality of the target species during the experiment are indicated in brackets. Four seedlings per block were needed to cover all species x treatment combinations. In cases where the total number of pots per species exceeded 24, seed material of more than one source was used. Transplanting depicts whether one or more timepoints were needed for transplanting; these were carried out between 22 May and 3 July 2018.

| **Species** | **Total** | **Block1** | **Block2** | **Block3** | **Block4** | **Block5** | **Block6** | **Transplanting date(s)** |
| --- | --- | --- | --- | --- | --- | --- | --- | --- |
| *Agrimonia eupatoria* | 24 | 4 | 4 | 4 | 4 | 4 | 4 | 05/22 |
| *Allium angulosum* | 24 | 4 | 4 | 4 | 4 | 4 | 4 | 06/05 |
| *Allium schoenoprasum* | 24 | 4 | 4 | 4 | 4 | 4 | 4 | 05/29 |
| *Allium suaveolens* | 24 | 4 | 4 | 4 | 4 | 4 | 4 | 05/29 |
| *Aquilegia atrata* | 26 | 4 | 4 | 4 | 6 | 4 (1) | 4 | 06/05, 06/12, 06/19, 07/03 |
| *Betonica officinalis* | 24 | 4 | 4 | 4 | 4 | 4 | 4 | 05/22 |
| *Carex davalliana* | 14 | 4 | 4 | 4 | 2 | 0 | 0 | 06/12 |
| *Carex distans* | 24 | 4 | 4 | 4 | 4 | 4 | 4 | 06/12 |
| *Carex flava* | 24 | 4 | 4 | 4 | 4 | 4 | 4 | 06/12, 07/03 |
| *Carex hostiana* | 3 | 0 | 3 | 0 | 0 | 0 | 0 | 06/19 |
| *Centaurium erythraea* | 24 | 4 | 4 | 4 | 4 | 4 | 4 | 05/29 |
| *Cirsium tuberosum* | 32 | 8 | 8 (1) | 4 | 4 | 4 | 4 | 05/22 |
| *Dianthus carthusianorum* | 24 | 4 | 4 | 4 | 4 | 4 | 4 | 05/22 |
| *Eriophorum latifolium* | 24 | 4 | 4 | 4 (1) | 4 (2) | 4 | 4 | 05/29 |
| *Galium boreale* | 24 | 4 | 4 | 4 | 4 | 4 | 4 | 05/29 |
| *Gentiana pneumonanthe* | 3 | 3 (1) | 0 | 0 | 0 | 0 | 0 | 06/12 |
| *Gratiola officinalis* | 24 | 4 | 4 (1) | 4 | 4 | 4 | 4 | 06/12, 07/03 |
| *Inula salicina* | 24 | 4 | 4 | 4 | 4 | 4 | 4 | 05/29 |
| *Iris sibirica* | 24 | 4 | 4 | 4 | 4 | 4 | 4 | 06/12 |
| *Juncus alpinus* | 24 | 4 | 4 | 4 | 4 | 4 | 4 | 06/12 |
| *Juncus subnodulosus* | 24 | 4 | 4 | 4 | 4 | 4 | 4 | 06/12 |
| *Linum catharticum* | 2 | 0 | 0 | 0 | 0 | 0 | 2 | 06/19 |
| *Lotus maritimus* | 24 | 4 | 4 | 4 | 4 | 4 | 4 | 05/22 |
| *Lotus pedunculatus* | 24 | 4 | 4 | 4 | 4 | 4 | 4 | 05/22 |
| *Lysimachia vulgaris* | 24 | 4 | 4 | 4 | 4 | 4 | 4 | 05/22 |
| *Menyanthes trifoliata* | 24 | 4 | 4 | 4 | 4 | 4 | 4 | 06/05, 06/12 |
| *Molinia caerulea* | 24 | 4 | 4 | 4 (1) | 4 | 4 | 4 | 05/29, 06/12 |
| *Ononis spinosa* | 24 | 4 | 4 | 4 | 4 | 4 | 4 | 05/22 |
| *Polygala amarella* | 15 | 0 | 2 | 4 (1) | 3 | 4 | 2 | 06/12, 06/19, 07/03 |
| *Potentilla erecta* | 24 | 4 | 4 | 4 | 4 (1) | 4 | 4 | 05/22 |
| *Primula farinosa* | 24 | 4 | 4 | 4 | 4 | 4 | 4 | 06/05 |
| *Ranunculus flammula* | 6 | 4 | 0 | 2 | 0 | 0 | 0 | 06/12, 06/19 |
| *Ranunculus nemorosus* | 24 | 4 | 4 | 4 | 4 | 4 | 4 (1) | 06/12 |
| *Sanguisorba officinalis* | 7 | 0 | 0 | 3 | 4 | 0 | 0 | 06/19, 07/03 |
| *Schoenus nigricans* | 6 | 0 | 0 | 4 | 0 | 2 | 0 | 06/19 |
| *Serratula tinctoria* | 24 | 4 | 4 | 4 | 4 | 4 | 4 (1) | 05/22 |
| *Succisa pratensis* | 24 | 4 | 4 | 4 | 4 | 4 | 4 | 05/29 |
| *Taraxacum sect. Palustria* | 24 | 4 | 4 | 4 | 4 | 4 | 4 | 05/22 |
| *Tofieldia calyculata* | 12 | 0 | 0 | 4 (2) | 4 (2) | 4(3) | 0 | 06/19 |
| *Triglochin palustris* | 48 | 8 | 8 | 8 | 8 | 8 | 8 | 05/29 |
| *Veronica scutellata* | 51 | 8 | 8 | 8 | 8 | 11 | 8 | 05/29, 06/12, 06/19, 07/03 |
| *Vincetoxicum hirundinaria* | 24 | 4 | 4 (1) | 4 | 4 | 4 | 4 | 06/05 |
| **Total** | 921 | 155 | 153 | 161 | 155 | 153 | 144 |  |

**Appendix Table S4.** Distribution of experimental pots over the six blocks and the representation within each block of the 42 target species and each of the four treatment combinations. Cases with mortality of the target species during the experiment are indicated in brackets. For information on partitioning of pots per species see Appendix Table S3.

| **Block** | **Pot number** | **Species number** | **Competition** | **Competition** | **No competition** | **No competition** |
| --- | --- | --- | --- | --- | --- | --- |
|  |  |  | **low-nutrient** | **high-nutrient** | **low-nutrient** | **high-nutrient** |
| B1 | 155 (1) | 36 | 39 | 39 | 39 (1) | 38 |
| B2 | 153 (3) | 36 | 38 (1) | 39 (1) | 38 | 38 (1) |
| B3 | 161 (5) | 39 | 40 (1) | 39 | 41 (4) | 41 |
| B4 | 155 (5) | 37 | 39 | 39 (2) | 39 (2) | 38 (1) |
| B5 | 153 (4) | 36 | 39 (1) | 39 | 37 (2) | 38 (1) |
| B6 | 144 (2) | 35 | 36 | 36 (2) | 36 | 36 |
| **Total** | 921 (20) | 42 | 231 (3) | 231 (5) | 230 (9) | 229 (3) |

**Appendix Table S5.** Standard deviations for individual target species random effects for all of the linear mixed models as provided applying the ‘varIdent’ function. Values are reported for the corresponding full linear mixed models using the restricted maximum-likelihood (REML) method. *Overall Species Standard and Residual Standard Deviation (Dev.) correspond to Species in Family output of the full model. The corresponding full model outputs are presented in Appendix Table S8 and Appendix Table S9, for results of likelihood-ratio tests (LRT) see Table 1 and Table 2.

|  | **All pots** |  | **Competition subset** |  | **No competition subset** | | | |
| --- | --- | --- | --- | --- | --- | --- | --- | --- |
| **Metric** | **above-ground biomass** |  | **target: total biomass** |  | **below-ground biomass** | **root-mass fraction** | **total root length** | **specific root length** |
| Transformation | ln |  | log 10 |  | log 10 | - | ln | ln |
|  | n=877 |  | n=442 |  | n=434 | n=433 | n=434 | n=433 |
| Species Standard Dev.* | 0.8714 |  | 0.3676 |  | 0.5315 | 0.1206 | 1.4091 | 0.5448 |
| Residual Standard Dev.* | 0.8721 |  | 0.3909 |  | 0.2835 | 0.0803 | 0.5621 | 0.2431 |
| *Agrimonia eupatoria* | 0.3324 |  | 0.3575 |  | 0.3293 | 0.4778 | 0.7556 | 1.5603 |
| *Allium angulosum* | 1.0000 |  | 1.0000 |  | 1.0000 | 1.0000 | 1.0000 | 1.0000 |
| *Allium schoenoprasum* | 1.0004 |  | 0.3994 |  | 0.5572 | 0.6451 | 0.7053 | 1.1478 |
| *Allium suaveolens* | 1.0838 |  | 0.7484 |  | 0.8950 | 1.3096 | 1.2216 | 1.1774 |
| *Aquilegia atrata* | 0.8090 |  | 0.8928 |  | 1.1254 | 0.7077 | 1.3363 | 1.2729 |
| *Betonica officinalis* | 0.5294 |  | 0.5903 |  | 0.8321 | 1.2324 | 0.7770 | 1.5826 |
| *Carex davalliana* | 0.4827 |  | 0.2769 |  | 0.6719 | 0.4725 | 1.0523 | 2.5446 |
| *Carex distans* | 0.4408 |  | 0.2814 |  | 0.2209 | 0.9148 | 0.6058 | 1.2877 |
| *Carex flava* | 0.8908 |  | 1.2506 |  | 0.4706 | 0.6865 | 0.5082 | 1.1641 |
| *Carex hostiana* | 0.8195 |  | 1.1009 |  | 0.0007 | 0.0004 | 0.0014 | 0.0012 |
| *Centaurium erythraea* | 0.8853 |  | 0.5870 |  | 1.4987 | 0.7015 | 1.8129 | 1.0851 |
| *Cirsium tuberosum* | 0.4185 |  | 0.4672 |  | 1.2165 | 1.8353 | 1.1544 | 2.5867 |
| *Dianthus carthusianorum* | 0.6310 |  | 0.3337 |  | 0.7519 | 0.9017 | 0.7465 | 1.3965 |
| *Eriophorum latifolium* | 1.4327 |  | 0.3121 |  | 1.8156 | 1.8319 | 1.8805 | 1.0164 |
| *Galium boreale* | 0.7403 |  | 0.9404 |  | 0.8992 | 1.0634 | 0.8606 | 0.9013 |
| *Gentiana pneumonanthe* | 1.5001 |  | 1.4307 |  | NA | NA | NA | NA |
| *Gratiola officinalis* | 0.7696 |  | 0.8436 |  | 0.8533 | 0.6183 | 1.3071 | 1.9100 |
| *Inula salicina* | 0.6592 |  | 0.6088 |  | 1.2558 | 0.9001 | 1.3077 | 1.2795 |
| *Iris sibirica* | 1.0974 |  | 0.5786 |  | 1.2086 | 0.6789 | 1.2967 | 1.4849 |
| *Juncus alpinus* | 0.8983 |  | 1.0669 |  | 0.4091 | 0.7034 | 0.6436 | 1.5198 |
| *Juncus subnodulosus* | 0.6868 |  | 0.8450 |  | 0.3207 | 0.2559 | 0.8456 | 1.9163 |
| *Linum catharticum* | 0.3328 |  | 0.3712 |  | NA | NA | NA | NA |
| *Lotus maritimus* | 1.0343 |  | 1.2906 |  | 0.5873 | 0.3807 | 0.7822 | 0.9692 |
| *Lotus pedunculatus* | 0.6330 |  | 0.5528 |  | 0.5947 | 1.1734 | 0.5349 | 0.8171 |
| *Lysimachia vulgaris* | 0.5705 |  | 0.6325 |  | 0.3540 | 0.3684 | 0.9774 | 2.6041 |
| *Menyanthes trifoliata* | 0.9034 |  | 0.8184 |  | 0.6368 | 0.5248 | 0.7102 | 0.6048 |
| *Molinia caerulea* | 0.8386 |  | 0.4969 |  | 1.9052 | 1.3367 | 1.7832 | 1.7052 |
| *Ononis spinosa* | 1.5336 |  | 1.4553 |  | 0.9784 | 1.2437 | 0.7368 | 1.3781 |
| *Polygala amarella* | 1.4075 |  | 0.5779 |  | 1.2568 | 1.1827 | 1.0568 | 2.5485 |
| *Potentilla erecta* | 1.2923 |  | 1.1971 |  | 0.2828 | 0.8000 | 0.6584 | 1.1219 |
| *Primula farinosa* | 0.6346 |  | 0.6100 |  | 0.8634 | 0.8160 | 1.2030 | 1.2240 |
| *Ranunculus flammula* | 0.5528 |  | 0.6384 |  | 0.6422 | 1.6379 | 0.2894 | 1.6430 |
| *Ranunculus nemorosus* | 1.6287 |  | 0.7737 |  | 1.5787 | 1.8849 | 1.8080 | 1.2491 |
| *Sanguisorba officinalis* | 0.8623 |  | 0.3023 |  | 0.8635 | 0.3964 | 1.0011 | 0.4575 |
| *Schoenus nigricans* | 0.6650 |  | 0.7192 |  | 0.1434 | 0.3188 | 0.7428 | 0.3397 |
| *Serratula tinctoria* | 0.5919 |  | 0.4757 |  | 0.6748 | 0.5649 | 1.0255 | 1.1478 |
| *Succisa pratensis* | 1.0830 |  | 0.6526 |  | 1.8141 | 1.0630 | 1.9661 | 0.8265 |
| *Taraxacum sect. Palustria* | 0.8443 |  | 0.7433 |  | 0.4072 | 0.4847 | 1.0357 | 2.1441 |
| *Tofieldia calyculata* | 6.1952 |  | 4.0312 |  | NA | NA | NA | NA |
| *Triglochin palustre* | 0.6781 |  | 0.6282 |  | 0.4564 | 0.4084 | 0.7010 | 1.3580 |
| *Veronica scutellata* | 0.9044 |  | 0.6798 |  | 1.7754 | 2.4295 | 1.9298 | 1.1325 |
| *Vincetoxicum hirundinaria* | 0.8721 |  | 0.4445 |  | 0.7612 | 2.4020 | 0.8425 | 1.0327 |

**Appendix Table S6.** Schematic overview of the hierarchical sequential removal of terms to determine the significance of fixed model terms by likelihood-ratio tests. The indicated term of interest was evaluated by comparing the likelihood of models with and without the term of interest (R) and all the terms indicated with a 1. Note that for testing terms including the 1911-Occurrence frequency (1911), the corresponding (interaction) term including “Index of change in occurrence frequency” was also excluded.

| **Above-ground biomass** | | | | | | | | | | | | | |
| --- | --- | --- | --- | --- | --- | --- | --- | --- | --- | --- | --- | --- | --- |
| Term of interest | **Model terms included in model** | | | | | | | | | | | | |
|  | **F:C:I** | **F:C:1911** | **F:I** | **F:1911** | **C:I** | **C:1911** | **F:C** | **F** | **C** | **I** | **1911** | **GT** | **IPH** |
| Fertilizer: Competition: Index of change in occurrence frequency (F:C:I) | R | 1 | 1 | 1 | 1 | 1 | 1 | 1 | 1 | 1 | 1 | 1 | 1 |
| Fertilizer: Competition: 1911 (F:C:1911) |  | R | 1 | 1 | 1 | 1 | 1 | 1 | 1 | 1 | 1 | 1 | 1 |
| Fertilizer: Index of change in occurrence frequency (F:I) |  |  | R | 1 | 1 | 1 | 1 | 1 | 1 | 1 | 1 | 1 | 1 |
| Fertilizer: 1911 (F:1911) |  |  |  | R | 1 | 1 | 1 | 1 | 1 | 1 | 1 | 1 | 1 |
| Competition: Index of change in occurrence frequency (C:I) |  |  | 1 | 1 | R | 1 | 1 | 1 | 1 | 1 | 1 | 1 | 1 |
| Competition: 1911 (C:1911) |  |  | 1 | 1 |  | R | 1 | 1 | 1 | 1 | 1 | 1 | 1 |
| Fertilizer: Competition (F:C) |  |  | 1 | 1 | 1 | 1 | R | 1 | 1 | 1 | 1 | 1 | 1 |
| Fertilizer (F) |  |  |  |  |  |  |  | R | 1 | 1 | 1 | 1 | 1 |
| Competition (C) |  |  |  |  |  |  |  | 1 | R | 1 | 1 | 1 | 1 |
| Index of change in occurrence frequency (I) |  |  |  |  |  |  |  | 1 | 1 | R | 1 | 1 | 1 |
| 1911-Occurrence frequency |  |  |  |  |  |  |  | 1 | 1 |  | R | 1 | 1 |
| Growing time (GT) |  |  |  |  |  |  |  |  |  |  |  | R | 1 |
| Initial plant height (IPH) |  |  |  |  |  |  |  |  |  |  |  | 1 | R |

**Appendix Table S7.** Schematic overview of the hierarchical sequential removal of terms to determine the significance of fixed model terms by likelihood-ratio tests. The indicated term of interest was evaluated by comparing the likelihood of models with and without the term of interest (R) and all the terms indicated with a 1. For all models presented here competition treatment was not applicable (dark grey shading) as the considered subsets included non-competition or competition only. Note that for models that had a tested term (R) with the 1911 Occurrence frequency, the corresponding (interaction) term including “Index of change in occurrence frequency” was mutually excluded.

| **below-ground biomass, root-mass fraction, specific root length,**  **total root length & ratio target: total above-ground biomass** | | | | | | | |
| --- | --- | --- | --- | --- | --- | --- | --- |
| Term of interest | **Model terms included in model** | | | | | | |
|  | **F:I** | **F:1911** | **F** | **I** | **1911** | **GT** | **IPH** |
| Fertilizer: Competition: Index of change in occurrence frequency (F:C:I) | Not applicable^a^ | | | | | | |
| Fertilizer: Competition: 1911 (F:C:1911) |  |  |  |  |  |  |  |
| Fertilizer: Index of change in occurrence frequency (F:I) | R | 1 | 1 | 1 | 1 | 1 | 1 |
| Fertilizer: 1911 (F:1911) |  | R | 1 | 1 | 1 | 1 | 1 |
| Competition: Index of change in occurrence frequency (C:I) | Not applicable^a^ | | | | | | |
| Competition: 1910 (C:1911) |  |  |  |  |  |  |  |
| Fertilizer: Competition (F:C) |  |  |  |  |  |  |  |
| Fertilizer (F) |  |  | R | 1 | 1 | 1 | 1 |
| Competition (C) | Not applicable^a^ | | | | | | |
| Index of change in occurrence frequency (I) |  |  | 1 | R | 1 | 1 | 1 |
| 1911-Occurrence frequency |  |  | 1 |  | R | 1 | 1 |
| Growing time (GT) |  |  |  |  |  | R^b^ | 1 |
| Initial plant height (IPH) |  |  |  |  |  | 1 | R^b^ |
| ^a^ Below-ground traits could not be obtained in the Competition treatment  ^b^ Note that the models for specific root-length and ratio target over total biomass metrics excluded Initial plant  height and Initial plant height and Growing time, respectively, due to convergence problems. | | | | | | | |

**Appendix Table S8.** Model summary statistics for linear mixed model analyses of the effect of nutrient addition and competition with *Poa pratensis* on above-ground target biomass, and the ratio of above-ground target biomass to combined above-ground biomass of target and competitor (*P. pratensis*). Model estimates are based on the output of the *lme* function fitted with restricted maximum-likelihood (REML) method.

|  | **ln (above-ground biomass)** | | | **log_10_ (target: total biomass ratio)** | | |
| --- | --- | --- | --- | --- | --- | --- |
| **Fixed effects** | **Estimate** | **Std. Error** | **t** | **Estimate** | **Std. Error** | **t** |
| Intercept | -3.2463 | 0.2384 | -13.62 | -1.7484 | 0.0998 | -17.53 |
| Initial plant height | 0.2617 | 0.0391 | 6.69 | 0.1284 | 0.0209 | 6.15 |
| Growing time | 0.0103 | 0.0050 | 2.06 | - | - | - |
| 1911 Occurrence frequency | -0.0980 | 0.0420 | -2.33 | -0.0444 | 0.0177 | -2.51 |
| Index of change in occurrence frequency^a^ | 0.3353 | 0.2764 | 1.21 | 0.1216 | 0.1148 | 1.06 |
| Competition (without *vs* with *Poa pratensis*) | 2.4641 | 0.0602 | 40.93 | - | - | - |
| Fertilizer (low-nutrient *vs* high-nutrient) | -0.3039 | 0.0590 | -5.15 | 0.0980 | 0.0211 | 4.64 |
| Fertilizer (low):Competition (without) | -0.2213 | 0.0842 | -2.63 | - | - | - |
| 1911:Competition (without) | 0.0375 | 0.0141 | 2.67 | - | - | - |
| 1911:Fertilizer (low) | -0.0179 | 0.0139 | -1.29 | 0.0027 | 0.0049 | 0.56 |
| Index of change in occurrence frequency: Competition (without) | -0.3948 | 0.1085 | -3.64 | - | - | - |
| Index of change in occurrence frequency: Fertilizer (low) | -0.1128 | 0.1083 | -1.04 | -0.0772 | 0.0353 | -2.19 |
| 1911:Competition (without):Fertilizer (low) | 0.0128 | 0.0197 | 0.65 | - | - | - |
| Index of change in occurrence frequency: Competition (without):Fertilizer (low) | 0.1912 | 0.1521 | 1.26 | - | - | - |
| **Random effects** | **Variance** | **Std. Dev.** | **Levels** | **Variance** | **Std. Dev.** | **Levels** |
| Family | 0.6532 | 0.8082 | 22 | 0.1148 | 0.3388 | 22 |
| Species within family^b^ | 0.7594 | 0.8714 | 42 | 0.1351 | 0.3676 | 42 |
| Residual | 0.7605 | 0.8721 |  | 0.1528 | 0.3909 |  |
| Number of observations |  |  | 877 |  |  | 442 |
| **R^2^ of the model** |  |  |  |  |  |  |
| Marginal R^2^ | 0.4310 |  |  | 0.1168 |  |  |
| Conditional R^2^ | 0.8009 |  |  | 0.6649 |  |  |
| ^a^ Index of change in occurrence frequency was calculated as the log-response ratio of the number of sites in which a species was present in  2017 relative to 1911.  ^b^ Standard deviations for the individual species’ random terms of the full model are shown in Appendix Table S5. | | | | | | |

**Appendix Table S9.** Model summary statistics for linear mixed model analyses of below-ground target biomass and root traits when grown without competition. Model estimates are based on the output of the *lme* function fitted with restricted maximum-likelihood (REML) method.

|  | **log_10_ (below-ground biomass)** | | | | **root-mass fraction** | | | **ln (total root length)** | | | | | **ln (specific root length)** | | |
| --- | --- | --- | --- | --- | --- | --- | --- | --- | --- | --- | --- | --- | --- | --- | --- |
| **Fixed effects** | **Estimate** | **Std. Error** | **t** | **Estimate** | | **Std. Error** | **t** | | **Estimate** | **Std. Error** | **t** | **Estimate** | | **Std. Error** | **t** |
| Intercept | -0.2184 | 0.1092 | -2.00 | 0.5578 | | 0.0213 | 26.19 | | 8.8768 | 0.2764 | 32.12 | 9.5229 | | 0.1052 | 90.50 |
| Initial plant height | 0.1043 | 0.0146 | 7.13 | 0.0106 | | 0.0046 | 2.32 | | 0.2106 | 0.0453 | 4.65 | NA | | NA | NA |
| Growing time | 0.0128 | 0.0026 | 4.88 | 0.0022 | | 0.0007 | 3.09 | | 0.0224 | 0.0058 | 3.87 | -0.0043 | | 0.0026 | -1.65 |
| 1911 Occurrence frequency | -0.0116 | 0.0239 | -0.48 | 0.0095 | | 0.0053 | 1.79 | | -0.0609 | 0.0625 | -0.97 | -0.0205 | | 0.0243 | -0.84 |
| Index of change in occurrence frequency^a^ | 0.0663 | 0.1495 | 0. | 0.0542 | | 0.0325 | 1.67 | | -0.0095 | 0.3892 | -0.02 | -0.1264 | | 0.1503 | -0.84 |
| Fertilizer (low-nutrient *vs* high-nutrient) | -0.1455 | 0.0152 | -9.58 | 0.0406 | | 0.0051 | 8.02 | | -0.2893 | 0.0456 | -6.35 | -0.0008 | | 0.0267 | -0.03 |
| 1911:Fertilizer (low) | -0.0003 | 0.0040 | -0.08 | -0.0008 | | 0.0014 | -0.60 | | 0.0215 | 0.0121 | 1.77 | 0.0099 | | 0.0071 | 1.40 |
| Index of change in occurrence frequency: Fertilizer (low) | 0.0029 | 0.0279 | 0.10 | -0.0225 | | 0.0082 | -2.74 | | -0.0097 | 0.0769 | -0.13 | -0.0577 | | 0.0382 | -1.51 |
|  |  |  |  |  | |  |  | |  |  |  |  | |  |  |
| **Random effects** | **Variance** | **Std. Dev.** | **Levels** | **Variance** | | **Std. Dev.** | **Levels** | | **Variance** | **Std. Dev.** | **Levels** | **Variance** | | **Std. Dev.** | **Levels** |
| Family | 0.0669 | 0.2587 | 20 | 0.0007 | | 0.0255 | 20 | | 0.3506 | 0.5921 | 20 | 0.0422 | | 0.2054 | 20 |
| Species within family^b^ | 0.2825 | 0.5315 | 39 | 0.0145 | | 0.1206 | 39 | | 1.9855 | 1.4091 | 39 | 0.2968 | | 0.5448 | 39 |
| Species Residual | 0.0804 | 0.2835 |  | 0.0064 | | 0.0803 |  | | 0.3160 | 0.5621 |  | 0.0591 | | 0.2431 |  |
| Number of observations |  |  | 434 |  | |  | 433 | |  |  | 434 |  | |  | 434 |
| **R^2^ of the model** |  |  |  |  | |  |  | |  |  |  |  | |  |  |
| Marginal R^2^ | 0.0792 |  |  | 0.1330 | |  |  | | 0.0523 |  |  | 0.0415 | |  |  |
| Conditional R^2^ | 0.8278 |  |  | 0.7417 | |  |  | | 0.8871 |  |  | 0.8577 | |  |  |
| ^a^ Index of change in occurrence frequency was calculated as the log-response ratio of the number of sites in which a species was present in 2017 relative to 1911.  ^b^ Standard deviations for the individual species’ random terms of the full model are shown in Appendix Table S5. | | | | | | | | | | | | | | | |
