# Supplementary material for: Declines in occurrence of plants characteristic for a nutrient‐poor meadow habitat are partly explained by their responses to nutrient addition and competition
Source: Ecol Evol. 2021 Mar 7;11(9):4058–70. doi: 10.1002/ece3.7306 (PMC8093689; doi:10.1002/ece3.7306)
Supplement: Supplementary file 4 — Supplementary Material [file ECE3-11-4058-s001.docx]

**Figure S1.** The effect of nutrient addition and competition with *Poa pratensis* on above-ground biomass production of 42 *Molinia-*meadow target species. Box plots show the mean of (a) the above-ground target biomass and (b) the ratio of target above-ground biomass to total above-ground biomass in pots with *P. pratensis* as competitor. Blue colors indicate competition with *P. pratensis*, and red colors indicate the absence of competition. Dark and light hues, respectively, indicate the high- and low-nutrient treatment. The y-axis depicts the back-transformed values on a log10 or ln-scale. The species are ranked according to increasing values of the index of change in occurrence frequency (log-response ratio of the 2017 relative to the 1911 occurrence frequency, for 14 intact *Molinia* meadows). For reference, species to the left of *Lysimachia vulgaris* (with an asterix) have declined, and species to the right have increased in occurrence frequency.

**Figure S2.** The effect of nutrient addition on below-ground biomass production and root traits of 42 *Molinia-*meadow target species when grown without competition. For high nutrient availability (dark red) and low nutrient availability (light red), box plots show the mean of (a) the total below-ground target biomass (log10-transformed), (b) the root-mass fraction, (c) the total root length (ln-transformed), and (d) the specific root length (ln-transformed). The species are ranked from left to right according to increasing values of the index of change in occurrence frequency (log-response ratio of the 2017 relative to the 1911 occurrence frequency, for 14 intact *Molinia* meadows). For reference, species to the left of *Lysimachia vulgaris* (with an asterix) have declined, and species to the right have increased in occurrence frequency.
